# Surveillance of *Vibrio parahaemolyticus* pathogens recovered from ready-to-eat foods

**DOI:** 10.1038/s41598-023-31359-4

**Published:** 2023-03-14

**Authors:** Abeni Beshiru, Etinosa O. Igbinosa

**Affiliations:** 1 Applied Microbial Processes and Environmental Health Research Group, Faculty of Life Sciences, University of Benin, Private Mail Bag 1154, Benin City, 300283 Edo State Nigeria; 2 Department of Microbiology, College of Natural and Applied Sciences, Western Delta University, Oghara, Delta State Nigeria; 3 Stellenbosch Institute for Advanced Study (STIAS), Wallenberg Research Centre at Stellenbosch University, Stellenbosch, South Africa

**Keywords:** Microbiology, Antimicrobials, Pathogens, Policy and public health in microbiology

## Abstract

This study examined the occurrence of *V. parahaemolyticus* from ready-to-eat (RTE) food in Delta State, Nigeria. It also characterized antibiotic resistance and virulence gene profile patterns to determine the associated health risk hazard. Food samples total of 380 were collected randomly and assessed for *V. parahaemolyticus*. *V. parahaemolyticus* isolates were characterized for their virulence and antibiogram potentials using a phenotypic and polymerase chain reaction (PCR) approach. A total of 42 (11.1%) samples were contaminated with *V. parahaemolyticus*. In 17/42 (40.5%) of the *V. parahaemolyticus*-positive samples, the densities were < 10 MPN/g. However, 19/42 (45.2%) and 6/42 (14.3%) of the samples had densities of 10 – 10^2^ and > 10^2^ MPN/g, respectively. A total of 67 V*. parahaemolyticus* isolates were identified using PCR; 54(80.6%) isolates were multidrug resistant. A total of 22 (32.8%), 39 (58.2%), and 67 (100%) of the *V. parahaemolyticus* harbored the *tdh*, *trh*, and *tlh* toxin genes, respectively. The T3SS1 gene (*vcrD1*) was detected in 67 (100%) of the isolates. The T3SS2α genes which were *vcrD2*, *vopB2*, and *vopT* were detected in 21 (31.3%), 11 (16.4%) and 30 (44.8%) of the isolates respectively. Some of the *V. parahaemolytics* strains harbored the *orf8* gene 20 (29.9%), and a combination of *orf8* + *tdh* genes 12 (17.9%), categorized as pandemic strains. The antibiotic resistance genes detected in this study include *bla*_TEM_ 33 (49.3), *tetM* 19 (28.4), *cmlA* 32(47.8) and *sul1* 14 (20.9). The concentration levels and prevalence of *V. parahaemolyticus* in RTE foods indicate contamination of ready-to-eat foods, particularly street foods consumed in the Delta State of Nigeria, threatening public health and consumer safety.

## Introduction

*Vibrio parahaemolyticus* (*V. parahaemolyticus*) is a gastroenteritis-causing food-borne pathogen, well-known as a Gram-negative bacterium common in food, food products and water bodies. It also secretes toxins that result in diarrheal illness. Recently in Nigeria, this pathogen has been recovered from processed seafood and African salads^1,2^. In other parts of the world, it has been isolated from various aquatic products and ready-to-eat (RTE) food^3,4^. In other African countries, there have been reports of *V. parahaemolyticus* from food^5–7^. However, there are limited studies on *V. parahaemolyticus* on its contamination levels and prevalence from processed RTE food in Nigeria, leading to decreased information on treatment strategies and monitoring in Nigeria. Therefore, this study provides data and in-depth insights specifying the significance of developing microbiological surveillance for *V. parahaemolyticus* strains as it affects food safety.

Food poisoning due to *V. parahaemolyticus* is usually linked with food consumption that is raw or has not been thoroughly cooked, improperly handled food that is cross-contaminated with the microorganism and its toxins^8^. Common symptoms include vomiting, diarrhoea, abdominal pain and mild fever, usually within one to two days of consumption of contaminated food. Implications like septicemia from individuals with *V. parahaemolyticus* infection can result in death^9^. The *tox*R gene is carried by *V. parahaemolyticus* strains, and it encodes a crucial membrane-situated regulatory protein that aids the regulation of the bacterial products expressed. The *tox*R expression can control the production of T3SS2, T3SS1, thermostable direct hemolysin (TDH) and TDH-related hemolysin (TRH)^10^. Some strains of *V. parahaemolyticus* from clinical origin do not possess TDH and TRH but remain pathogenic with different strategies employed by different strains^11^. To date, *tdh*, *tox*R, and *trh* genes have been detected in *V. parahaemolyticus* isolates using polymerase chain reaction (PCR)^2,12^.

Antibiotics application has contributed immensely to animal and human treatment since the discovery of penicillin. Increasing research has shown that the increased prevalence of *V. parahaemolyticus* with antibiotic-resistant (AR) potentials may substantially threaten economic and public health development for humans globally^13^. Due to the excessive application of antibiotics in food animals and humans in the last few years, antibiotic resistance has evolved in *V. parahaemolyticus*. Some *V. parahaemolyticus* isolates from foods and other food products are frequently resistant to antibiotics such as chloramphenicol, aminoglycosides (gentamicin and streptomycin), ampicillin, ciprofloxacin, among others^2,9^. The potential of RTE foods to harbor AR *V. parahaemolyticus* can present a significant public health menace linked to managing and controlling the disease. *Vibrio parahaemolyticus* recovered from various sources has shown resistance to single or multiple antibiotics^1^. Multidrug resistance (MDR) in *V. parahaemolyticus* directly affects antibiotics application, prevention and treatment regimens^14^. Hence, it is imperative to institute an efficient monitoring mechanism to aid treatment regimens of the *V. parahaemolyticus* with clinically relevant antibiotics.

The United Nations General Assembly in 2018 adopted resolution 73/250 proclaiming June 7 as World Food Safety Day, taking into account the global burden of food-borne diseases which affect people of all age grades, especially children < 5 years and persons living in low-income regions^15^. The World Health Organization (WHO) estimated that 420,000 persons die yearly globally from consuming contaminated foods. The fatalities are from an estimated 600 million people falling sick from eating food contaminated by bacteria, viruses, or chemical substances^15^. There are > 200 diseases, ranging from diarrhoea to cancers^15^. There is no food security without food safety. Hence, concerted efforts must be made to reduce the number of deaths and cases recorded annually.

Food-borne diseases are the major international challenge that remains under-discussed, which makes the situation poorly understood, especially its impacts on people’s health and economies. Food contamination with antibiotic-resistant bacteria (ARB) can lead to its transfer to humans, a global health concern. There is an increasing focus on treating life-threatening infections caused by *Vibrio* pathogens that typically exhibit antibiotic resistance. In Nigeria, 200,000 people die of food poisoning each year, according to the National Agency for Food and Drug Administration and Control^16^. There is a need for Nigeria to achieve Sustainable Development Goals (SDGs) by 2030. Food safety underlines many of the goals, including SDG 2 (zero hunger), SDG 3 (good health and well-being), and SDG 8 (decent work and economic growth). Food safety contributes to achieving the SDGs and is a cross-cutting area. Poor food safety practices harm families' health, workforce capacity, tourism, healthcare systems, and the economy. This necessitates regulatory agencies of the food safety industry to diligently implement policy measures and legal frameworks to strengthen the national food safety system and ensure it complies with food safety standards. RTE food is widely consumed in Nigeria, and proper hygiene is essential. This study examined the occurrence of *V. parahaemolyticus* from RTE food in Delta State, Nigeria. It also characterized antibiotic resistance and virulence gene profile patterns to determine the associated health risk hazard.

## Results

### Biochemical, prevalence and levels of *V*. *parahaemolyticus* contamination from RTE foods

*V. parahaemolyticus* isolates from this study were Gram-negative curved rods, motile with one polar flagellum, oxidase-positive, negative for methyl-red and Voges-Proskauer test, proliferated in tubes of nutrient broth containing 8% NaCl, urease positive, grew on T_1_N_0_ and T_1_N_3_ media, negative to ortho-nitrophenyl-ꞵ-galactoside, positive to 3.5% NaCl triple-sugar-iron test, negative to arginine hydrolyzation. In addition, the isolates were positive for d-mannitol, d-mannose, and arabinose utilization but negative for lactose fermentation and variable for d-cellobiose fermentation.

Of the 380 RTE food samples obtained from Delta State, Nigeria, 42 (11.1%) samples were positive for *V. parahaemolyticus*. In 17/42 (40.5%) of the *V. parahaemolyticus*-positive samples, the densities were < 10 MPN/g. However, 19/42 (45.2%) and 6/42 (14.3%) of the samples had densities of 10 – 10^2^ and > 10^2^ MPN/g respectively (Table 1). The prevalence based on the food outlets studied includes fast food restaurants 4/89 (4.49%), cafeterias 7/103 (6.79%), and street food 31/188 (16.49%). Values with statistical differences carry different alphabets across columns (Table 1). *P*-values less than 0.05 were considered statistically different A total of 67 *V. parahaemolyticus* isolates were identified using PCR via specific primers and further characterized.

**Table 1:** Prevalence levels of *V.* *parahaemolyticus* contamination from RTE foods.

Food sample type	N^o^ of the analyzed samples	N^o^ of samples positive	Levels of samples contaminated in MPN/g (*n* = 42)		
			< 10	10 − 10^2^	> 10^2^ – 10^3^
Banga soup	19	4 (21.1)^cd^	–	3 (7.1)^bc^	1 (2.4)^a^
Starch	19	–	–	–	–
Jollof rice	19	5 (26.3)^de^	2 (4.8)^ab^	3 (7.1)^ab^	–
Fish pepper soup	19	1 (5.3)^a^	1 (2.4)^a^	–	–
Egusi soup	19	4 (21.1)^cd^	1 (2.4)^a^	2 (4.8)^ab^	1 (2.4)^a^
Ogbono soup	19	1 (5.3)^a^	1 (2.4)^a^	–	–
Owo soup	19	2 (10.5)^ab^	1 (2.4)^a^	1 (2.4)^a^	–
Coconut rice	19	2 (10.5)^ab^	1 (2.4)^a^	1 (2.4)^a^	–
Fried rice	19	5 (26.3)^de^	2 (4.8)^ab^	2 (4.8)^ab^	1 (2.4)^a^
Goat meat pepper soup	19	–	–	–	–
White melon pepper soup	19	1 (5.3)^a^	1 (2.4)^a^	–	–
Agidi jollof	19	3 (15.8)^bc^	1 (2.4)^a^	2 (4.8)^ab^	–
Egg sauce	19	–	–	–	–
Iribotor	19	1 (5.3)^a^	1 (2.4)^a^	–	–
White ukodo	19	2 (10.5)^ab^	–	2 (4.8)^ab^	–
Oil ukodo	19	–	–	–	–
Owo beans soup	19	3 (15.8)^bc^	2 (4.8)^ab^	1 (2.4)^a^	–
Obiokpo soup	19	2 (10.5)^ab^	1 (2.4)^a^	1 (2.4)^a^	–
Black soup	19	–	–	–	–
Vegetable soup	19	6 (31.6)^e^	2 (4.8)^ab^	1 (2.4)^a^	3 (7.1)^b^
Total	380	42 (11.1)	17 (40.5)	19 (45.2)	6 (14.3)

Values with statistical differences carry different alphabets across columns.

### Antimicrobial susceptibility profile of the* V. parahaemolyticus *isolates

The antimicrobial resistance (AMR) profile of the *V. parahaemolyticus* isolates in Table 2 (Supplementary Table 3) includes ampicillin 38 (56.7%), tetracycline 37 (55.2%), chloramphenicol 36 (53.7%), trimethoprim-sulfamethoxazole 28 (41.8%), ciprofloxacin 24 (35.8%), cefotaxime 21 (31.3%), nalidixic acid 19 (28.4%), azithromycin 19 (28.4%), ceftazidime 17 (25.4%), streptomycin 11 (16.4%) and ampicillin/sulbactam 13 (19.4%). The antimicrobials to which the isolates were most commonly susceptible to includes: imipenem 67 (100%), gentamicin 65 (97.0%), azithromycin 40 (59.7%), ampicillin/sulbactam 43 (64.2%), streptomycin 40 (59.7%), nalidixic acid 37 (55.2%), cefotaxime 32 (48.8%) and ceftazidime 31 (46.3%).

**Table 2:**
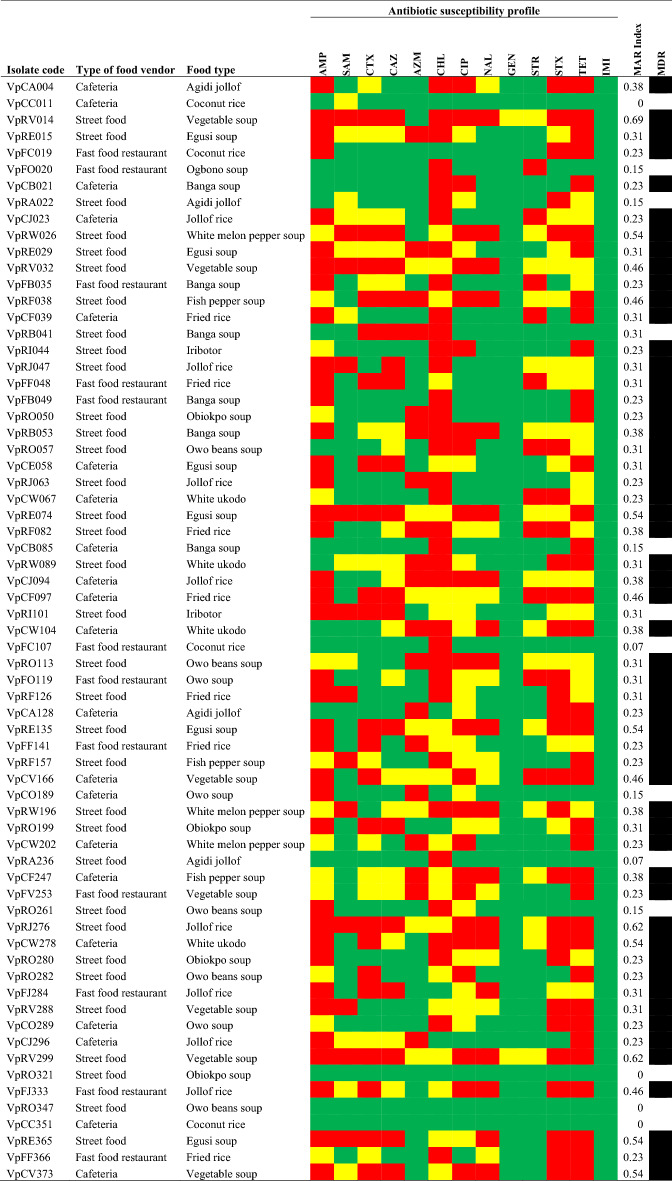
Distribution profile of *V.* *parahaemolyticus* antibiotic susceptibility.

Red filled square = resistance, green filled square = sensitive, yellow filled square = intermediate, black filled square = positive, white filled square = negative.

*MDR* multidrug resistance, *MAR* multiple antibiotic resistance, *AMP* ampicillin, *CAZ* ceftazidime, *CTX* cefotaxime, *SAM* ampicillin/sulbactam, *AZM* azithromycin, *CIP* ciprofloxacin, *CHL* chloramphenicol, *STR* streptomycin, *NAL* nalidixic acid, *STX* trimethoprim-sulfamethoxazole, *TET* tetracycline, *IMI* imipenem, *GEN* gentamicin.

### Multiple antibiotic resistance index (MARI) and multidrug resistance (MDR) profile of the *V*. *parahaemolyticus* isolates

The MARI based on the retail food outlets includes cafeterias (0 – 0.54), street food (0 – 0.69), and fast-food restaurant (0.07 – 0.46). Most isolates with MARI > 0.4 were recovered from street food samples (Table 2). A total of 12/67 (17.9%) *V. parahaemolyticus* isolates had MARI < 0.2 while 55/67 (82.1%) had MARI > 0.2. A total of 54 (80.6%) were MDR, while 13 (19.4%) were not MDR (Table 2). No isolate was extensively drug-resistant or pan-drug resistant. Three of the isolates were sensitive to all antibiotics used. A total of 63 (94.0%) of the isolates were resistant to ≥ 1 antimicrobial drug used (Table 2). Isolate VpRV299 (from vegetable soup from street food) and VpRJ276 (from Jollof rice from street food) had a MAR index of 0.62. The highest MAR index was recorded from VpRV014 (from vegetable soup from street food), with a MAR index of 0.69 (Table 2).

### Antimicrobial resistance (AMR) genes profile in *V*. *parahaemolyticus* isolates

**Figure 1:**
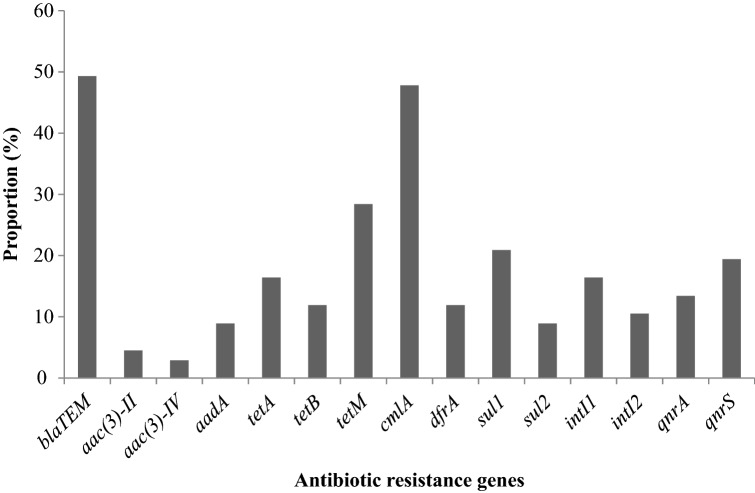
The proportion of antibiotic resistance genes distribution pattern of *V.* *parahaemolyticus*.

The antibiotic resistance genes detected in Fig. 1 in this study include *bla*_TEM_ 33(49.3), *aac(3)-II* 3(4.5), *aac(3)-IV* 2(2.9), *aadA* 6(8.9), *tetA* 11(16.4), *tetB* 8(11.9), *tetM* 19(28.4), *cmlA* 32(47.8), *dfrA* 8(11.9), *sul1*14(20.9), *sul2* 6(8.9), *intI1* 11(16.4), *intI2* 7(10.5), *qnrA* 9(13.4), and *qnrS* 13(19.4). Antimicrobial resistance genes such as *bla*_SHV_, *bla*_OXA_, *tetC*, *floR*, *sul3*, *qnrB* and *qnrC* were undetected. All 33 *bla*_TEM_-positive isolates were resistant to ampicillin, whereas 13 of the isolates were also resistant to ampicillin/sulbactam (Fig. 1). Nine streptomycin-resistant isolates were positive for aminoglycoside genes screeded (*aac(3)-II* = 3, *aadA* = 6). Two intermediate gentamicin-resistant isolates were positive for the *aac(3)-IV* genes. Of the 37 isolates resistant to tetracycline, 11 were *tetA* positive, eight were *tetB* positive, and 19 were *tetM* positive. Three of the 19 tetM-positive isolates were simultaneously positive for the *tetB* genes (Fig. 1). All 32 isolates positive for the *cmlA* genes were phenotypically positive for the chloramphenicol antibiotics. Six of the isolates that were positive for the *dfrA* genes were resistant to the trimethoprim-sulfamethoxazole antibiotics, while the other two isolates showed intermediate resistance phenotypically (Fig. 1).

The isolates harbored between 0 and 8 resistance genes screened. The isolate with the highest (8) antibiotic resistance gene was VpRV014. It was recovered from street food (Vegetable soup) (Fig. 1). All six *sul2* isolates were resistant to trimethoprim-sulfamethoxazole antibiotics, while from the 14 isolates positive for *sul1*, 12 were resistant to trimethoprim-sulfamethoxazole while 2 showed intermediate phenotypic resistance. All the *qnrA* positive isolates and 6/13 *qnrS* positive isolates were phenotypically resistant to ciprofloxacin, while 7/13 of the *qnrS* positive isolates were phenotypically resistant to nalidixic acid. All the 11 and 7 isolates that were positive for class I and II integrons were MDR. The class I integron-positive isolates had a MAR index between 0.38 and 0.69, harboring between 3 and 8 AMR genes. Class II integron-positive isolates had a MAR index between 0.46 and 0.62 and carried between 4 and 6 AMR genes (Fig. 1).

### Extracellular virulence factors formation of *V*. *parahaemolyticus* isolates

**Figure 2:**
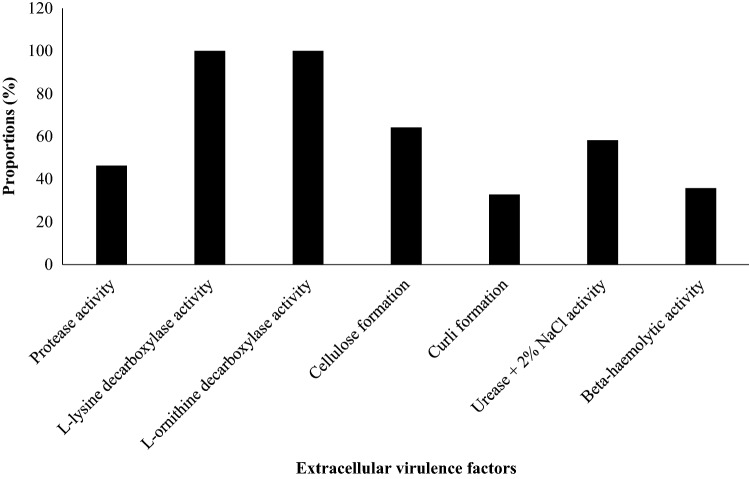
Extracellular virulence factors formation of *V.* *parahaemolyticus* isolates.

The extracellular virulence characteristics of the isolates in Fig. 2 include protease activity 31 (46.3%), l-lysine decarboxylase activity 67 (100%), l-ornithine decarboxylase activity 67 (100%), cellulose formation 43 (64.2%), curli formation 22(32.8%), urease + 2% NaCl activity 39 (58.2%) and beta-hemolytic activity 24 (35.8%). All the isolates were negative for l-arginine decarboxylase activity. Protease activity significantly correlated cellulose formation (*r* = 0.506, *p* < 0.01), curli formation (*r* = 0.690, *p* < 0.01), urease + 2% NaCl activity (*r* = 0.604, *p* < 0.01), beta hemolytic activity (*r* = 0.493, *p* < 0.01), *tdh* (*r* = 0.562, *p* < 0.01), *trh* (*r* = 0.604, *p* < 0.01), *vcrD2* (*r* = -0.304, *p* < 0.05), and *orf8* (*r* = 0.376, *p* < 0.01). (*r* = , *p* < 0.01), (*r* = , *p* < 0.01), (*r* = , *p* < 0.01), (*r* = , *p* < 0.01) (Table 3).

**Table 3:** Correlation of phenotypic and genotypic virulence of *V.* *parahaemolyticus* strains.

	MARI	PA	CF	CUF	UN	BHA	*tdh*	*trh*	*vcrD2*	*vopB2*	*vopT*	*orf8*
MARI	1.000**											
PA	0.700**	1.000										
CF	0.698**	0.506**	1.000									
CUF	0.791**	0.690**	0.522**	1.000								
UN	0.642**	0.604**	0.629**	0.592**	1.000							
BHA	0.575**	0.493**	0.363**	0.604**	0.507**	1.000						
*Tdh*	0.617**	0.562**	0.456**	0.662**	0.592**	0.936**	1.000					
*Trh*	0.642**	0.604**	0.629**	0.592**	1.000**	0.507**	0.592**	1.000				
*vcrD2*	− 0.006	− 0.304*	0.169	− 0.130	− 0.080	− 0.169	− 0.198	− 0.080	1.000			
*vopB2*	0.106	− 0.171	0.090	− 0.049	− 0.022	− 0.248*	− 0.224	− 0.022	0.410**	1.000		
*vopT*	0.339**	0.188	0.360**	0.201	0.215	0.204	0.137	0.215	0.492**	0.105	1.000	
*orf8*	0.437**	0.376**	0.351**	0.377**	0.354**	0.329**	0.377**	0.354**	− 0.089	− 0.200	0.200	1.000

*MARI* multiple antibiotic resistance index, *PA* protease activity, *CF* cellulose formation, *CUF* curli formation, *UN* urease + 2% NaCl activity, *BHA* beta hemolytic activity.

**correlation is significant at the 0.01 level (2-tailed), *correlation is significant at the 0.05 level (2-tailed).

Cellulose formation significantly correlated curli formation (*r* = 0.522, *p* < 0.01), urease + 2% NaCl activity (*r* = 0.629, *p* < 0.01), beta hemolytic activity (*r* = 0.363, *p* < 0.01), *tdh* (*r* = 0.456, *p* < 0.01), *trh* (*r* = 0.629, *p* < 0.01), *vopT* (*r* = 0.360, *p* < 0.05), and *orf8* (*r* = 0.351, *p* < 0.01). Curli formation significantly correlated urease + 2% NaCl activity (*r* = 0.592, *p* < 0.01), beta hemolytic activity (*r* = 0.604, *p* < 0.01), *tdh* (*r* = 0.662, *p* < 0.01), *trh* (*r* = 0.592, *p* < 0.01), and *orf8* (*r* = 0.377, *p* < 0.01). Urease + 2% NaCl activity significantly correlated beta hemolytic activity (*r* = 0.507, *p* < 0.01), *tdh* (*r* = 0.592, *p* < 0.01), *trh* (*r* = 1.000, *p* < 0.01), and *orf8* (*r* = 0.354, *p* < 0.01). Beta hemolytic activity significantly correlated *tdh* (*r* = 0.936, *p* < 0.01), *trh* (*r* = 0.507, *p* < 0.01), *vopB2* (*r* = − 0.248, *p* < 0.05) and *orf8* (*r* = 0.329, *p* < 0.01) (Table 3).

### Virulence genes detected in *V*. *parahaemolyticus* isolates

**Figure 3:**
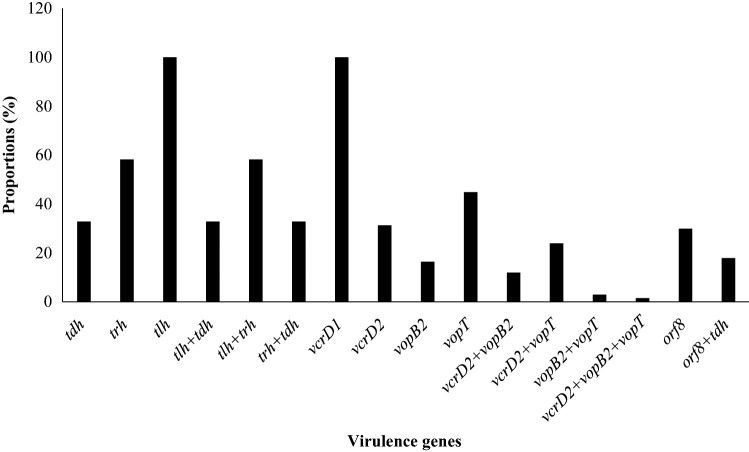
Virulence genes detected in *V.* *parahaemolyticus* isolates.

A total of 22 (32.8%), 39 (58.2%), and 67(100%) of the *V. parahaemolyticus* harbored the *tdh*, *trh*, and *tlh* toxin genes, respectively. In addition, 22 (32.8%), 39 (58.2%), and 22 (32.8%) of the *V. parahaemolyticus* harbored a combination of toxin genes such as *tlh* + *tdh*, *tlh* + *trh*, and *trh* + *tdh* respectively (Fig. 3). The T3SS1 gene: *vcrD1* was detected in 67 (100%) of the isolates. The T3SS2α genes: *vcrD2*, *vopB2*, and *vopT* were detected in 21 (31.3%), 11 (16.4%) and 30(44.8%) of the isolates respectively. A combination of T3SS2α genes: *vcrD2* + *vopB2*, *vcrD2* + *vopT* and *vopB2* + *vopT* were detected in 8 (11.9%), 16 (23.9%), 2 (2.9%) of the isolates respectively. Some of the *V. parahaemolytics* strains harbored the *orf8* gene 20 (29.9%) and a combination of *orf8* + *tdh* genes 12 (17.9%) as such, were categorized as pandemic strains (Fig. 3). The isolates had ≥ 2 and ≤ 7 virulence genes (Fig. 1). The two isolates with the highest number of virulence genes were VpCW067 and VpRV299. VpRV299 was recovered from street food (from Vegetable soup), while VpCW067 was recovered from a cafeteria (from white “ukodo”). Significant correlations exist between isolates that possessed the *orf8* genes and MAR index (*r* = 0.437, *p* < 0.01), protease activity (*r* = 0.376, *p* < 0.01), cellulose formation (*r* = 0.351, *p* < 0.01), curli formation (*r* = 0.377, *p* < 0.01), urease + 2% NaCl activity (*r* = 0.354, *p* < 0.01), beta-hemolytic activity (*r* = 0.329, *p* < 0.01), *tdh* (*r* = 0.377, *p* < 0.01), *trh* (*r* = 0.354, *p* < 0.01) (Table 3). The *tdh* gene significantly correlated *trh* (*r* = 0.592, *p* < 0.01) and *orf8* (*r* = 0.377, *p* < 0.01). The *trh* gene significantly correlated *orf8* (*r* = 0.354, *p* < 0.01). The *vcrD2* gene significantly correlated *vopB2* (*r* = 0.410, *p* < 0.01) and *vopT* (*r* = 0.492, *p* < 0.01) (Table 3).

## Discussion

Food-borne pathogens can enter the human intestinal tract and cause disease, making them essential for understudying *V. parahaemolyticus* pathogenesis. The overall cell density of *V. parahaemolyticus* by Li et al.^4^ ranged between 1.50 and 100 MPN/g; 64.36% of samples had a bacterial density of 3 to 10 MPN/g, 18.81% of samples had bacterial density > 3 MPN/g, and 16.83% samples exceeded 10 MPN/g^4^. Li et al.^4^’s results were lower than ours. The cell density counts of *V. parahaemolyticus* by Igbinosa et al.^2^ varied between 1.5 and 1000 MPN/g which was related to the outcomes of this study. From the positive samples by Igbinosa et al.^2^, 21.21% had bacterial density < 3 MPN/g, 39.39% had 3–10 MPN/g, 27.27% had > 10–100 MPN/g, and 12.12% samples had > 100–1,000 MPN/g. The difference between our study and those of Igbinosa et al.^2^ from Nigeria was the type of food examined. Our study examined different kinds of RTE foods, while the study of Igbinosa et al.^2^ focused on African salad. Although the study of Li et al.^4^ from China examined RTE foods, there was a disparity in the type of RTE food studied due to cultural, traditional and geographical differences. To prevent food-borne diseases, it is indispensable to pinpoint the necessity for assessing microbiological risks of food and improve active monitoring efforts, mainly geared towards hygienic management.

The isolation of several *V. parahaemolyticus* strains from RTE foods designated environmental contamination, poor handling methods, and pitiable hygiene. Microbial contamination of food products post-handling can result from unclean hands, unhygienic food preparation procedures, meagre water quality, and materials for washing utensils. Prevalence between 3.67 and 4.96% compared to our study has been reported^4,17^. A higher prevalence between 37.5 and 90% has also been documented^18–20^. Several outbreaks of *V. parahaemolyticus* from food in different countries happen through the ingestion of food. This has become a key global threat to human health and food safety. Many other locally processed indigenous foods are consumed frequently as street foods. Hence, *V. parahaemolyticus* is a probable cause of infectious illness in Delta State, Nigeria.

The prevalence of AMR *V. parahaemolyticus* in RTE food is a severe concern to human health and food safety globally. *V. parahaemolyticus* infections are mostly self-limiting; however, severe or prolonged infectious disease requires antibiotic therapy. Most isolates from this study showed resistance to streptomycin, ampicillin, nalidixic acid, tetracycline, ciprofloxacin, ampicillin/ sulbactam, cefotaxime, ceftazidime, chloramphenicol, trimethoprim-sulfamethoxazole, and azithromycin; which are similar to other studies^13,21,22^. The penicillin group is the most prescribed antibiotic for the primary treatment of food-borne illnesses in Nigeria partly due to its cost-effectiveness compared to others. The development of penicillin-resistant bacteria dwindled its efficacy. Resistance to ampicillin was reportedly higher among *Vibrio* species^2,18^ compared to those from our study. Ampicillin and ampicillin-sulbactam resistance (100%) reported previously^9,20,23^ contradicted ours. The possible rationale for lower ampicillin resistance could be attributed to the different food types, and the hygiene studied. Though the ampicillin resistance in this study was 56.7%, it was the antibiotics to which the isolates from this study were most resistant. Higher levels of cephalosporin resistance were also reported previously^19,20^ compared to findings from our research. Lei et al.^17^ revealed that isolates were susceptible to ceftazidime and cefotaxime, signifying third-generation cephalosporins effective toward *V. parahaemolyticus* infections.

Findings on tetracycline resistance from our study were higher than previously reported^2,17^. Higher levels of quinolone and fluoroquinolone resistance were observed in our study than previously reported^19,20^. Additionally, the isolates from our study exhibited lower resistance to aminoglycosides compared to previous reports^2,4^. *V. parahaemolyticus* can give and take genetic elements in favorable environments, enhancing antibiotic resistance. The primary mechanisms of resistance to fluoroquinolone are the existence of plasmid-mediated quinolone resistance (PMQR) genes and mutations in the quinolone resistance-determining regions (QRDRs)^24^. Findings from our study underscore the significance of proper antibiotic monitoring and usage to safeguard food. To overcome MDR, antibiotic substitutes are required to treat and prevent disease and ensure food sustainability and quality. All fluoroquinolone-resistant *V. parahaemolyticus* by Lei et al.^17^ showed MAR to other clinically relevant antimicrobials, similar to our study's findings. This represents a public health threat as these antimicrobials are CDC recommended for Vibrio species treatment^12^.

Our data showed that 80.6% of *V. parahaemolyticus* had a phenotype of MDR higher than previously reported^4,25^. In addition, MAR index values > 0.2 designate high-risk sources for AMR contamination, which poses a significant threat to public health. This also implies probabilities of intrinsic resistance to antimicrobial drugs. The high stability of AR bacteria in RTE foods indicates a potential risk to food products. The high levels of intermediate susceptibility to antimicrobial drugs portray possible resistance to such drugs. The MAR index from our study ranged from 0 to 0.69, which is lower and contrasts the findings from previous studies^2,23^ and is similar to earlier reports^20^. Ahmed et al.^23^ reported that all isolates showed resistance to ≥ 7 antibiotics, which is way above our study's findings. A total of 82.1% of isolates from our study had a MAR index > 2 which is higher than previous studies^18,26^. Although the highest MAR index value of 0.82 was reported by Ashrafudoulla et al.^26^, 0.69 was reported as the highest in our study. The different MAR index reported can be ascribed to divergent routes of samples studied, geographic distribution, and the method used.

*V. parahaemolyticus* isolates by Lei et al.^17^ that were fluoroquinolone-resistant harbored PMQR genes, which is consistent with the results from our study as all the *qnrA* and *qnrS* positive isolates were phenotypically resistant to quinolone antibiotics. This contrasted with the results of Liu et al.^27^. Evolution and mutation of resistance genes are essential for emerging MDR strains and thus play crucial roles in the treatment regimen. MDR isolates by Igbinosa et al.^2^ possessed resistant genes similar to our findings. The *dfr* resistance gene detection from our study levels with previous studies^1,2^.

Lower proportions of the *bla*_TEM_ gene were recovered from our study compared to others^22,28^. A higher proportion of resistance genes (*sul3*, *sul2*, *sul1*, *tetM*, *bla*_*TEM*_) were detected by Igbinosa et al.^2^ compared to those from our study with the exemption of *sul3* gene that was not detected from our research. *bla*_*OXA*_ and *tetB* determinants were not documented in previous studies^2,29^, but *bla*_*TEM*_ and *tetB* genes were observed in our findings. Resistance genes such as *tetA* and *bla*_*TEM*_ by Jeamsripong et al.^30^ were common to genes from our study. Results from our study showed diverse resistance determinants in multi-resistant pathogenic *Vibrio* strains from RTE food. Therefore, these pathogenic and pandemic clones with integron determinants may be instrumental as acceptors and donors of transmissible AMR elements. Resistance elements inside integrons aid an effective mechanism of disseminating AR amongst bacteria from diverse ecosystems. The *bla*_*TEM*_ gene and other antibiotic resistance determinants in *Vibrio* strains designate transferring these elements into the environment.

A 2% concentration of NaCl can result in osmotic stress on ordinary intracellular activities but be endured by *Vibrio* strains due to its halophilic potential. Sangadkita et al.^31^ reported *E. coli* O157:H7 inactivation when cultivated on TSB + 8.5% NaCl concentration, yet *Vibrio* strains were not inactivated. The result on hemolytic and protease activities validates previous results^8^. Hemolytic activity by *V. parahaemolyticus* increases its nutrient utilization from host cells, thereby increasing its virulence. This is problematic as 35.8% *V. parahaemolyticus* isolates from this study showed β-haemolytic activity. In addition, *V. parahaemolyticus* isolates from this study expressed extracellular proteases, which have been reported to facilitate the breakdown of cellular components of the host matrix, disrupt cellular signalling to short-circuit practices and improve bacterial persistence to hash cellular conditions^32^. Such occurrences portray danger to RTE foods, directly impacting consumer safety. Bacterial persistence in food processing plants has been reported as a transmission route for the widespread pathogenic superbugs. The curli and cellulose produced by *V. parahaemolyticus* in this study have shown previous potentials in bacterial biofilm structure, adherence and persistence. The curli confers bacteria with the essential prowess to surface adhere and improve its pathogenic characteristics^33^. Isolates that couldn’t possess adherence characteristics possessed fewer alleles and genes than those characterized with such prowess^3^, similar to our study's findings. All isolates revealed significant genetic and adherence characteristics on mussel and shrimp surfaces^26^, higher than our study’s. *V. parahaemolyticus* isolates formed strong adherence characteristics on the non-living surface^34^, which was in line with the findings from our study as curli and cellulose, which as essential biofilm components, were expressed. Differences in adherence characteristics can be ascribed to structural component differences such as adhesive surface proteins, pili and fimbriae^26^. Studying the phenotypic and extracellular virulence activity of *V. parahaemolyticus* is crucial to food safety and human health as it aids the proper understanding of *V. parahaemolyticus* with a pathway for mitigating the pathogenesis and survival of the pathogens.

Our study revealed that *the isolates amplified tlh and toxR entirely*, similar to previous studies^22,28,34^. Likewise, findings for T3SS1 and T3SS2 genes have been documented^22^. Previous reports showed that only 0.2–10% of *V. parahaemolyticus* harbor the *tdh* and *trh* toxin elements^21,23^. The *toxR* gene from our study has been documented to be involved in gene regulation, such as biofilm formation, bacterial persistence, and other virulence attributes^35^. The *tdh, trh* and *tlh* are determinants of the hemolysin gene crucial in *V. parahaemolyticus* pathogenesis. The products of *tdh* and *trh* from *V. parahaemolyticus* in this study are believed to induce inflammatory gastroenteritis^1,8,32^ rapidly. Thus, detecting the hemolysin genes could be an essential way to infer the virulence potential of food poisoning. Abd-Elghany and Sallam^36^ reported *tdh* was detected in 7.4% *V. parahaemolyticus* from seafood assessed with two isolates from shrimp specimens having the *trh* determinants. The *tdh* gene was not detected in all crustacean samples evaluated by Ahmed et al.^23^, with 8.4% of isolates positive for *tdh* and *trh* determinants. Some studies have reported a complete lack of *trh* and *tdh* genes in *V. parahaemolyticus* from food^14,37^, contrary to our research findings. Food contaminated with *trh* and *tdh* genes induces inflammatory gastroenteritis^38^.

Our results agree with previous data where urease production correlates with *trh-* and *tdh* -positive *V. parahaemolyticus* isolates^2,13^; however, this contradicts Sujeewa et al.’s report^39^. Narayanan et al.^18^ reported 29/648 (4.8%) isolates exhibited β-hemolytic activity, whereas 17/29 β-hemolytic positive isolates were negative for the *trh* gene but amplified the *tdh* gene. Only 1.6% of the samples tested positive for β-hemolysis by Abd-Elghany and Sallam^36^, with 16% of the negative hemolytic isolates harboring the *tdh* determinants. The absence or presence of virulence determinants in *V. parahaemolyticus* may depend on the variations in geographical regions, sample sources and testing methodologies, which explain the disparity of gene prevalence when comparing our study to others. For instance, 20.7% of samples harbored the *tdh* gene from the southwest coast of India after 18 h enrichment in bile salt sodium taurocholate (ST) broth by PCR^25^. In another study, 19% of samples harbored *tdh* by enrichment using ST broth followed by colony hybridization^25^, whereas there was 100% *tdh* detection after 24 h enrichment in LB broth followed by PCR from the mussel samples from the west coast area of Korea^26^. The *tdh* gene was reported positive in 55 environmental samples by MPN-PCR procedure, whereas no *the*-carrying *Vibrio* strain was recovered by the MPN-culture method^40^. Considering that RTE foods are prevalent in Nigeria and do not often require additional heat treatment or curing before eating (mainly from the street foods), the prevalence of *V. parahaemolyticus* in these foods, and the presence of virulence genes in these isolates, impose a huge threat to food safety and public health.

The virulence gene screened by Lei et al.^38^ revealed that 44 (27.00%) *Vibrio* strains had ≥ 1 virulence gene (lower when compared to our study). Virulence proteins can be obtained via horizontal gene transmission, which boosts the bacterium's fitness in an environment. The *orf8*, *vcrD1*, *vcrD2*, *vopB2* and *vopT* genes recovered from isolates in this study have aberrant base compositions and are crucial in pandemic clones’ evolution^26^. The findings reflect the potential of food in the study area for disseminating pandemic strains of *V. parahaemolyticus* and the suitability of pandemic determinants for detecting pandemic strains in samples of food origin. The occurrence of *orf8 V. parahaemolyticus* strains from RTE foods in this study signifies the pandemic clones, which are detrimental to public health due to their enhanced virulence capabilities. *V. parahaemolyticus* strains from this study encoded T3SS1 (*vcrD1*, *vcrD2*), which allows the death of the infected host cells, leading to the discharge of essential nutrients. This curtailed virulence attribute from these isolates, especially from RTE foods, calls for concern. T3SS1 and T3SS2α (*vopB2* and *vopT*) genes recovered from *V. parahaemolyticus* isolates in this study have been reported to be pivotal in vesicular transport, secretion and intracellular trafficking, which can translate to the translocation of cellular proteins and actin crosslinking^26,34^. Such crucial pathogenic characteristics can undermine the safety and quality of RTE foods in the study region.

Our results concurred with the results of Myers et al.^41^; thus, the *orf8* determinant can be instrumental as a marker for detecting pandemic *Vibrio* strains. Analysis by Pang et al.^3^ showed that *V. parahaemolyticus* from RTE food possessed more genes than other environmental (water and aquatic product) and clinical isolates. Ashrafudoulla et al.^26^ reported that 87.5% of *Vibrio* strains harboring the T3SS. Findings from our study showed RTE food in the region as a probable cradle of pandemic and pathogenic *Vibrio* strain, reflecting the public health menace of consuming not adequately cooked or mishandled foods. Quercetin, eugenol, marine-derived secondary metabolites, bubble technique, cold plasma, ultrasound, Ultraviolet-C spectrum, high hydrostatic pressure, photodynamic inactivation, curcumin-based photosensitization, small-molecule signal blocker, enzymes and bacteriophages have also found application in the control of food-borne pathogens^42,43^.

## Conclusion

This study provides in-depth evidence that locally processed indigenous RTE foods in Delta State Nigeria are a potential reservoir of pathogenic *V. parahaemolyticus*, thus posing a human health risk, especially for Nigerians who enjoy street food consumption. The pandemic clones suggest RTE foods as a source of *Vibrio* infections due to their virulence and multiple antimicrobial resistance potential and thus portray a public health concern on food safety and quality. The multiple antibiotic resistance index recovered from the study depicts inappropriate use of antibiotics in the study area culminating in the development and widespread of superbugs. This study's findings further support the possible dissemination of *V. parahaemolyticus* and its resistant determinants and its usefulness in designing microbiological risk assessment models to estimate the incidence of *V. parahaemolyticus* in RTE foods. A clear monitoring plan in Nigeria is necessary to assess the genuine public health risk comprehensively.

## Materials and methods

### Sample collection

The study exclusively focused on RTE foods from Delta State, Nigeria. The sample size determination formula was used to determine the sample size as follows:

$$\mathrm{Sample }\left(\mathrm{N}\right)=\frac{{({\mathrm{Z}}_{1-\propto /2})}^{2}\mathrm{ P}(1-\mathrm{P}) }{{d}^{2}}.$$

Z_1-α/2_ = Standard regular variant at 5% type I error (P < 0.05); P = Expected prevalence founded on a previous study [3.67%^4^; 36.2%^14^; 43.75%^44^; 5.9%^37^]; d = Absolute error or precision (which is 5%). A total of 380 RTE food samples were collected randomly from Warri, Sapele and Oghara food outlets, all in Delta State, Nigeria, from July 2021 to February 2022, using sterile universal containers for sample collection. The sample distribution from the food outlets includes fast food restaurants (*n* = 89), cafeterias (*n* = 103), and street food (*n* = 188). More samples were collected from street food, the most prevalent and patronized in the region, followed by the cafeteria. All the samples were sealed and conveyed in an ice box to the laboratory for analysis within six hours (6 h) of sample collection.

### Enumeration and isolation of *V*. *parahaemolyticus* from the RTE food samples

Fifty (50) grams of RTE food samples were weighed up and homogenized into a sterile 450 mL of alkaline peptone water (Lab M, Lancashire, United Kingdom), pH 8.5 with 2% NaCl added, giving a first-order dilution (1:10). The samples were homogenized for 1 min at 800 rpm using a shaker. A 3 × 10 mL ration of the 1:10 dilution was inoculated into three tubes comprising 10 mL of double-strength alkaline peptone water. Likewise, 3 × 1 mL portions of the 1:100, 1:1000 and 1:10,000 dilutions were inoculated into 10 mL of single-strength alkaline peptone water and incubated for 24 h at 35 ± 2 °C^45^. The streak plate method via a loopful from the top 1 cm of the alkaline peptone water tubes having the highest dilutions of the sample presenting turbidity was inoculated onto thiosulphate-citrate-bile salt-sucrose (TCBS) agar (Lab M, Lancashire, United Kingdom) and incubated at 35 ± 2 °C for 24 h. *V. parahaemolyticus* appear 2–3 mm in diameter, round, bluish-green or green, opaque colonies on TCBS agar. *V. parahaemolyticus* colonies were expressed in MPN/g. Colonies selected for identification ranged from ≥ 1 –  ≤ 5. *Vibrio paraheamolyticus* DSM 11,058 was used as a positive control. Isolates were purified on tryptone soy agar (Lab M, Lancashire, United Kingdom) supplemented with 2% NaCl and stored in nutrient agar (Lab M, Lancashire, United Kingdom) slants containing 2% NaCl at 4 °C until ready for further use.

### Morphological and biochemical characterizations of the *V*. *parahaemolyticus* isolates

*V. parahaemolyticus* isolates from this study were screened for Gram reaction and motility test. Other biochemical tests include oxidase, methyl-red, Voges-Proskauer, growth on 8% NaCl, urease; growth on tryptone salt broths (T_1_N_0_ and T_1_N_3_); ortho-nitrophenyl-ꞵ-galactoside, 3.5% NaCl triple-sugar-iron test, arginine hydrolyzation. In addition, the *V. parahaemolyticus* isolates were screened for sugar fermentation^45^.

### Molecular identification of the *V*. *parahaemolyticus* isolates

#### Genomic DNA extraction

The genomic DNA was extracted following the method of Chen and Kuo^46^ with modifications. Briefly, 3.0 mL of an overnight culture was grown in Luria–Bertani (LB) broth at 37 °C for 16 h and centrifuged at 27,787×*g* for 3 min. The pellet was transferred into 200µL of lysis buffer (40 mM, pH 7.8, 20 mM sodium acetate, Tris–acetate, 1 mM EDTA, 1% SDS), mixed gently and incubated for 30 min at 37 °C. A 50 µL of 5 M NaCl suspension was centrifuged at 27,787 × *g* for 10 min. Followed by mixing the supernatant with 200µL of chloroform and centrifuging for 10 min at 27,787 × *g*. DNA from the upper aqueous phase was precipitated with 200 µL isopropanol, washed with 70% ethanol, dried and re-suspended in 50 µL TE buffer (Tris/EDTA buffer with RNase) for PCR. The DNA was stored at − 20 °C until used.

### PCR detection, virulence and antibiogram characterization of *V*. *parahaemolyticus* isolates

For the amplification of the genomic DNA, primers in Supplementary Table 1 (Table S1) were used to identify and characterize the *V.* *parahaemolyticus* for its antibiotic resistance and virulence determinants. *Vibrio* *paraheamolyticus* DSM 11,058 was used as a positive control. The 50 μl PCR cocktail includes: 10 μl of gDNA, 5 μl PCR buffer with MgCl_2_, 2.5 μl F primer (adjusted to 10 pmol/μl), 2.5 μl R primer (adjusted to 10 pmol/μl), 6 μl dNTP mix, 0.3 μl Taq polymerase and 23.7 μl nuclease-free water, were introduced into the appropriate PCR-tubes. All PCR mixtures were pipetted up and down to ensure proper mixing. After brief centrifugation, the PCR tubes were placed in the wells of the Peltier-Based Thermal Cycler. The individual steps of the PCR program for the amplification of the DNA are as follows: initial denaturation for 3 min at 94 °C, annealing to the primer used in Supplementary Table 1 (Table S1), extension for 1.3 min at 72 °C, and a final extension for 5 min at 72 °C for 32 cycles. For gel electrophoresis, 1.0% agarose gel comprised 4 g agarose and 1 × 400 ml TAE buffer. The gel was run for 1 h at 100 V DC voltage.

### Virulence factor formation

All isolates were inoculated on blood agar containing 10% sheep blood respectively and incubated for 24 h at 37 °C. Clear hemolysis and discoloration of the blood medium to pale yellow indicated a positive hemolysis test. The formation of curli and cellulose was determined as described previously^33^. Briefly, curli and cellulose formation were assayed using tryptone soy agar (Lab M, Lancashire, United Kingdom) supplemented with 40 mg of Congo red (Sigma) per liter and 20 mg of brilliant blue (Sigma) per liter. Isolates were plated onto Congo red plates and incubated for 48 h at 28 °C before determining morphotypes. Isolates were grouped into three distinct morphotypes: (i) rough, dry, and red, indicating cellulose and curli formation (rdar); (ii) rough, dry, and brown, indicating curli formation but a lack of cellulose synthesis (bdar); and (iii) white and smooth, indicating a lack of both cellulose and curli formation (saw).

For the decarboxylase test, a 5 μl of 18–24 h brain heart infusion broth (Merck, Darmstadt Germany) culture was incorporated into each of the three decarboxylase broths (l-ornithine, l-lysine, and l-arginine) with a control tube included. A 4 mm film of sterile mineral oil was added to the respective tubes and incubated at 35–37 °C in ambient air for 4 d. The tubes were observed for change of color at 24, 48, 72, and 96 h. A positive test was a turbid purple to faded-out yellow-purple color (alkaline). The extracellular protease of the isolates was assayed on TSA plates supplemented with 1% casein (v/v). Colonies grown on tryptone soy agar (TSA) were suspended in 3 ml of Mueller–Hinton broth. The density of this suspension was adjusted to 0.5 McFarland standards, equivalent to 10^6^ cells/mL. A 5 mL sample of this suspension was inoculated on TSA plates supplemented with 1% casein and incubated at 37 °C for 24 to 48 h. The zone of clearance due to casein hydrolysis was considered a positive result.

### Antimicrobial susceptibility profiling

The antimicrobial susceptibility profiling of the *V. parahaemolyticus* isolates was done using the Kirby-Bauer disc diffusion method and interpreted by adopting the breakpoints of the Clinical and Laboratory Standard Institute^47^. Briefly, purified isolates were inoculated on 5 mL TSB and incubated overnight. A total of 13 antibiotic discs (Mast Diagnostics, Merseyside, United Kingdom) which includes penicillins [ampicillin/sulbactam (10/10 μg), ampicillin (10 μg)], aminoglycosides [gentamicin (10 μg), streptomycin (10 μg)], carbapenems [imipenem (10 μg)], cephalosporins [cefotaxime (30 μg), ceftazidime (30 μg)], quinolones [ciprofloxacin (5 μg), nalidixic acid (30 μg)], phenicols [chloramphenicol (30 μg)], folate pathway inhibitor [trimethoprim-sulfamethoxazole (1.25/23.75 μg)], tetracyclines [tetracycline (30 μg)] and macrolides [azithromycin (15 μg)] were employed. The antimicrobials were chosen based on their relevance clinically to *V. parahaemolyticus* infections. *Vibrio paraheamolyticus* DSM 11,058 was used as a positive control. The susceptibility profile of the isolates was compared with the interpretative chart to determine the intermediate, resistant and sensitive nature of the isolates^47^; Supplementary Table 2). The multiple antibiotic resistance index (MARI) was calculated as described^48^, with MAR index > 0.2 characterized as high risk while MAR index < 0.2 were characterized as low risk. MDR profile was estimated as described^49^.

$$\mathrm{Multiple \,\,antibiotic \,\, resistance  \,\,index}= \frac{\mathrm{Number  \,\,of \,\, the \,\, antibiotics \,\, to  \,\,which \,\, resistance \,\, occurred}}{\mathrm{Total  \,\,number \,\, of  \,\,antibiotics \,\, to  \,\,which \,\, the \,\, isolates \,\, were \,\, tested}}.$$

### Data analysis

The data were examined for correctness and totality. Analysis was carried out via SPSS statistical software version 20 (IBM Corp, USA). Expressive prevalence statistics were presented in frequency tables, mean, percentages and standard deviations with their corresponding 95% Confidence intervals. The prevalence data were expressed via appropriate cross-tabulations. The significant level was set at a *p-value* < 0.05.

## Supplementary Information

Supplementary Information.

## Data Availability

All data generated or analyzed during this study are included in this published article (and its Supplementary Information files). Other information/data related to the current study are available from the corresponding author on reasonable request.
