# Supplementary material for: Surveillance of Vibrio parahaemolyticus pathogens recovered from ready-to-eat foods
Source: Sci Rep. 2023 Mar 14;13:4186. doi: 10.1038/s41598-023-31359-4 (PMC10011769; doi:10.1038/s41598-023-31359-4)
Supplement: Supplementary file 1 — Supplementary Information. [file 41598_2023_31359_MOESM1_ESM.docx]

For submission as an article in **Scientific Reports**

Supplementary File

**Surveillance of *Vibrio parahaemolyticus* pathogens recovered from ready-to-eat foods**

Abeni Beshiru^1,2,3^, and Etinosa O. Igbinosa^1,3^*

^1^Applied Microbial Processes and Environmental Health Research Group, Faculty of Life Sciences, University of Benin, Private Mail Bag 1154 Benin City, 300283 Edo State Nigeria

^2^ Department of Microbiology, College of Natural and Applied Sciences, Western Delta University, Oghara, Delta State Nigeria

^3^ Stellenbosch Institute for Advanced Study (STIAS), Wallenberg Research Centre at Stellenbosch University, Stellenbosch, South Africa

***** Corresponding author E-mail: Etinosa.Igbinosa@uniben.edu (EOI)

**Table S1.** Primers used in this study

| **Target genes** | **Primer sequence (**5’- 3’**)** | **Amplicon**  **size (bp)** | **Annealing**  **condition** | **References** |
| --- | --- | --- | --- | --- |
| Toxin operon gene  (*tox*-R) | *toxR*-F (ATACGAGTGGTTGCTGTCATG)  *toxR*-R (GTCTTCTGACGCAATCGTTG) | 368 | 68°C for  1 min | [1] |
| Thermostable direct  hemolysin (*tdh*) | *tdh*-F (GTAAAGGTCTCTGACTTTTGGAC)  *tdh*-R (TGGAATAGAACCTTCATCTTCACC) | 270 | 58°C for  1 min | [2] |
| TDH-related  hemolysin (*trh*) | *trh*-F (TTGGCTTCGATATTTTCAGTATCT)  *trh*-R (CATAACAAACATATGCCCATTTCCG) | 486 | 58°C for  1 min | [2] |
| Thermolabile  hemolysin (*tlh*) | *tlh*-F (AAAGCGGATTATGCAGAAGCACTG)  *tlh*-R (TGTGCCTTGATGAACTCGTTC) | 170 | 60 °C for  1 min | [3] |
| Adhesive protein  (*orf8*) | *orf8*-F (AGGACGCAGTTACGCTTGATG)  *orf8*-R (CTAACGCATTGTCCCTTTGTAG) | 369 | 53 °C for  1 min | [4] |
| *Vibrio* calcium-  response protein (*vcrD1*) | *vcrD1*-F (CTGCTGGTCTTGTTCGCTCT)  *vcrD1*-R (TCTGGTCGCTTCCTTCTGTG) | 493 | 53 °C  for 1 min | [5] |
| *Vibrio* calcium-  response protein  (*vcrD2*) | *vcrD2*-F (GTTGGTGCTCGCTTCTCTCT)  *vcrD2*-R (CCCATCCCCTACTGTCAAGA) | 300 | 53 °C  for 1 min | [5] |
| Translocation  protein (*vopB2*) | *vopB2*-F (GGGGGCAAGCTAATAAAGAGAT)  *vopB2*-R (GTTAAAGCTGAGCAACATCGTG) | 527 | 53 °C  for 1 min | [5] |
| Effector protein  (*vopT*) | *vopB2*-F (GTGAAGGTTTGTAGAATACATACGGAAA)  *vopB2*-R (TCACTTAGCTAAATCTAGCGCATC) | 690 | 53 °C  for 1 min | [6] |
| Beta-lactams  (*bla*_TEM_) | TEM-F (ATTCTTGAAGACGAAAGGGC)  TEM-R (ACGCTCAGTGGAACGAAAAC) | 1150 | 60 °C  for 1 min | [7] |
| Beta-lactams  (*bla*_SHV_) | SHV-F (CACTCAAGGATGTATTGTG)  SHV-R (TTAGCGTTGCCAGTGCTCG) | 885 | 52 °C  for 1 min | [8] |
| Beta-lactams  (*bla*_OXA_) | OXA-F (ACACAATACATATCAACTTCGC)  OXA-R (AGTGTGTTTAGAATGGTGATC) | 813 | 61 °C  for 1 min | [9] |
| Aminoglycosides  (*aac(3)-II*) | AacC2-F (ACTGTGATGGGATACGCGTC)  AacC2-R (CTCCGTCAGCGTTTCAGCTA) | 237 | 60 °C  for 1 min | [10] |
| Aminoglycosides  (*aac(3)-IV*) | AacC4-F (CTTCAGGATGGCAAGTTGGT)  AacC4-R (TCATCTCGTTCTCCGCTCAT) | 286 | 60 °C  for 1 min | [10] |
| Aminoglycosides  (*aadA*) | AadA-F (GCAGCGCAATGACATTCTTG)  AadA-R (ATCCTTCGGCGCGATTTTG) | 282 | 60 °C  for 1 min | [11] |
| Tetracyclines  (*tetA*) | TetA-F (GTAATTCTGAGCACTGTCGC)  TetA-R (CTGCCTGGACAACATTGCTT) | 937 | 62 °C  for 1 min | [12] |
| Tetracyclines  (*tetB*) | TetB-F (CTCAGTATTCCAAGCCTTTG)  TetB-R (CTAAGCACTTGTCTCCTGTT) | 416 | 57 °C  for 1 min | [12] |
| Tetracyclines  (*tetC*) | TetC-F (TCTAACAATGCGCTCATCGT)  TetC-R (GGTTGAAGGCTCTCAAGGGC) | 570 | 62 °C  for 1 min | [12] |
| Tetracyclines  (*tetM*) | TetM-F (GTGGACAAAGGTACAACGAG)  TetM-R (CGGTAAAGTTCGTCACACAC) | 406 | 61 °C  for 1 min | [13] |
| Chloramphenicols  (*cmlA*) | CmlA-F (TGTCATTTACGGCATACTCG)  CmlA-R (ATCAGGCATCCCATTCCCAT) | 455 | 55 °C  for 1 min | [14] |
| Chloramphenicols  (*floR*) | FloR1 (CACGTTGAGCCTCTATAT)  FloR2 (ATGCAGAAGTAGAACGCG) | 868 | 55 °C  for 1 min | [15] |
| Trimethoprim  (*dfrA*) | DfrIa-F (GTGAAACTATCACTAATGG)  DfrIa-R (TTAACCCTTTTGCCAGATTT) | 474 | 55 °C  for 1 min | [16] |
| Sulphonamides  (*sul1*) | Sul-F (TGGTGACGGTGTTCGGCATTC)  Sul-R (GCGAGGGTTTCCGAGAAGGTG) | 789 | 63 °C  for 1 min | [17] |
| Sulphonamides  (*sul2*) | Sul2-F (CGGCATCGTCAACATAACC)  Sul2-R (GTGTGCGGATGAAGTCAG) | 722 | 50 °C  for 1 min | [18] |
| Sulphonamides  (*sul3*) | Sul3-F (CATTCTAGAAAACAGTCGTAGTTCG)  Sul3-R (CATCTGCAGCTAACCTAGGGCTTTGGA) | 990 | 51 °C  for 1 min | [19] |
| Class I integron  (*intI1*) | IntI1-F (GGGTCAAGGATCTGGATTTCG)  IntI1-R (ACATGGGTGTAAATCATCGTC) | 483 | 62 °C  for 1 min | [17] |
| Class II integron  (*intI2*) | IntI2-F CACGGATATGCGACAAAAAGGT  IntI2-R GTAGCAAACGAGTGACGAAATG | 788 | 62 °C  for 1 min | [17] |
| Quinolones  (*qnrA*) | QnrA-F (GATCGGCAAAGGTTAGGTCA)  QnrA-R (ATTTCTCACGCCAGGATTTG) | 516 | 55 °C  for 1 min | [20] |
| Quinolones  (*qnrB*) | QnrB-F (GATCGTGAAAGCCAGAAAGG)  QnrB-R (ACGATGCCTGGTAGTTGTCC) | 469 | 57 °C  for 1 min | [20] |
| Quinolones  (*qnrC*) | QnrC-F (GGGTTGTACATTTATTGAATCG)  QnrC-R (CACCTACCCATTTATTTTCA) | 307 | 56 °C  for 1 min | [20] |
| Quinolones  (*qnrS*) | QnrS-F (ACGACATTCGTCAACTGCAA)  QnrS-R (TAAATTGGCACCCTGTAGGC) | 417 | 56 °C  for 1 min | [20] |

**Table S2.** Antibiotic susceptibility breakpoints of the *V. parahaemolyticus*

| **Antimicrobial class** | **Antibiotics** | ***V. parahaemolyticus* CLSI breakpoints (mm)** | | |
| --- | --- | --- | --- | --- |
|  |  | **Resistance** | **Intermediate** | **Sensitive** |
| Penicillins | Ampicillin/sulbactam (10/10 μg) | ≤11 | 12-14 | ≥15 |
|  | Ampicillin (10 μg) | ≤13 | 14-16 | ≥17 |
| Aminoglycosides | Gentamicin (10 μg) | ≤12 | 13-14 | ≥15 |
|  | Streptomycin (10 μg) | ≤11 | 12-14 | ≥15 |
| Carbapenems | Imipenem (10 μg) | ≤19 | 20-22 | ≥23 |
| Cephalosporins | Cefotaxime (30 μg) | ≤22 | 23-25 | ≥26 |
|  | Ceftazidime (30 μg) | ≤17 | 18-20 | ≥21 |
| Quinolones | Nalidixic acid (30 μg) | ≤13 | 14-18 | ≥19 |
|  | Ciprofloxacin (5 μg) | ≤15 | 16-20 | ≥21 |
| Phenicols | Chloramphenicol (30 μg) | ≤12 | 13-17 | ≥18 |
| Folate pathway inhibitor | Trimethoprim-sulfamethoxazole (1.25/23.75 μg) | ≤10 | 11-15 | ≥16 |
| Tetracyclines | Tetracycline (30 μg) | ≤11 | 12-14 | ≥15 |
| Macrolides | Azithromycin (15 μg) | ≤13 | 14-17 | ≥18 |

**Table S3.** Occurrence of antimicrobial susceptibility profile of the *V. parahaemolyticus* isolates

| **Antimicrobial class** |  | ***V. parahaemolyticus* isolates (*n*=67)** | | |
| --- | --- | --- | --- | --- |
|  | **Antibiotics** | **Resistance** | **Intermediate** | **Sensitive** |
| Penicillins | Ampicillin/sulbactam | 13(19.4) | 11(16.4) | 43(64.2) |
|  | Ampicillin | 38(56.7) | 14(20.9) | 15(22.4) |
| Aminoglycosides | Gentamicin | 0 | 2(2.9) | 65(97.0) |
|  | Streptomycin | 11(16.4) | 16(23.9) | 40(59.7) |
| Carbapenems | Imipenem | 0 | 0 | 67(100) |
| Cephalosporins | Cefotaxime | 21(31.3) | 14(20.9) | 32(48.8) |
|  | Ceftazidime | 17(25.4) | 19(28.4) | 31(46.3) |
| Quinolones | Nalidixic acid | 19(28.4) | 11(16.4) | 37(55.2) |
|  | Ciprofloxacin | 24(35.8) | 22(32.8) | 21(31.3) |
| Phenicols | Chloramphenicol | 36(53.7) | 21(31.3) | 10(14.9) |
| Folate pathway inhibitor | Trimethoprim-sulfamethoxazole | 28(41.8) | 17(25.4) | 22(32.8) |
| Tetracyclines | Tetracycline | 37(55.2) | 20(29.9) | 10(14.9) |
| Macrolides | Azithromycin | 19(28.4) | 8(11.9) | 40(59.7) |

**REFERENCES**

1. Kim, Y. B. *et al*. Identification of *Vibrio parahaemolyticus* strains at the species level by PCR targeted to the *tox*R gene. *J. Clin. Microbiol.* **37**, 1173-1177 (1999).
2. Bej, A. K. *et al*. Detection of total and hemolysin-producing *Vibrio parahaemolyticus* in shellfish using multiplex PCR amplification of *tl*, *tdh* and *trh*. *J. Microbiol. Method.* **36**, 215–225 (1999).
3. Rizvi, A. V. & Bej, A. K. Multiplexed real-time PCR amplification of *tlh*, *tdh* and *trh* genes in *Vibrio parahaemolyticus* and its rapid detection in shellfish and Gulf of Mexico water. *Antonie Van Leeuwenhoek* **98**, 279–290 (2010).
4. Myers, M. L., Panicker, G. and Bej, A. K. PCR detection of a newly emerged pandemic *Vibrio parahaemolyticus* O3:K6 pathogen in pure cultures and seeded waters from the Gulf of Mexico. *Appl. Environ. Microbiol.* **69**, 2194–2200 (2003).
5. Tsai, S. E. *et al*. Molecular characterization of clinical and environmental *Vibrio parahaemolyticus* isolates in Taiwan. *Int. J. Food Microbiol.* **165**, 18–26 (2013).
6. Kodama, T. *et al*. Identification and characterization of *VopT*, a novel ADP-ribosyltransferase effector protein secreted via the *Vibrio parahaemolyticus* type III secretion system 2. *Cell. Microbiol.* **9**, 2598–2609 (2007).
7. Belaaouaj, A. *et al*. Nucleotide sequences of the genes coding for the TEM-like -lactamases IRT-1 and IRT-2 (formerly called TRI-1 and TRI-2). *FEMS Microbiol. Lett*. **120**, 75–80 (1994).
8. Pitout, J. D. D. *et al*. Lactamases responsible for resistance to expanded-spectrum cephalosporins in *Klebsiella pneumoniae, Escherichia coli*, and *Proteus mirabilis* isolates recovered in South Africa. *Antimicrob. Agents Chemother*. **42**, 1350–1354 (1998).
9. Steward, C. D. *et al*. Characterization of clinical isolates of *Klebsiella pneumoniae* from 19 laboratories using the National Committee for Clinical Laboratory Standards extended-spectrum -lactamase detection methods. *J. Clin. Microbiol*. **39**, 2864–2872 (2001).
10. van de Klundert, J. A. M. & Vliegenthart, J. S. PCR detection of genes coding for aminoglycoside-modifying enzymes. In D. H. Persing, T. F. Smith, F. C. Tenover, and T. J. White (ed.), Diagnostic molecular microbiology. American Society for Microbiology, Washington, D.C. p. 547–552 (1993).
11. Madsen, L., Aarestrup, F. M. & Olsen. J. E. Characterisation of streptomycin resistance determinants in Danish isolates of *Salmonella* Typhimurium. *Vet. Microbiol*. **75**, 73–82 (2000).
12. Guardabassi, L., Dijkshoorn, L., Collard, J. M., Olsen, J. E. & Dalsgaard, A. Distribution and *in-vitro* transfer of tetracycline resistance determinants in clinical and aquatic *Acinetobacter* strains. *J. Med. Microbiol*. **49**, 929–936 (2000).
13. Ng, L. K., Martin, I., Alfa, M. & Mulvey, M. Multiplex PCR for the detection of tetracycline resistant genes. *Mol. Cell. Probes* **15**, 209–215 (2001).
14. Saenz, Y. *et al*. Mechanisms of resistance in multiple-antibiotic-resistant *Escherichia coli* strains of human, animal, and food origins. *Antimicrob. Agents Chemoth*. **48**(10), 3996–4001 (2004).
15. Ng, L. K., Mulvey, M. R., Martin, I., Peters, G. A. & Johnson, W. Genetic characterization of antimicrobial resistance in Canadian isolates of *Salmonella* serovar Typhimurium DT104. *Antimicrob. Agents Chemother*. **43**, 3018–3021 (1999).
16. Navia, M. M., Ruiz, J. Sanchez-Cespedes, J. & Vila, J. Detection of dihydrofolate reductase genes by PCR and RFLP. *Diagn. Microb. Infect. Dis*. **46**, 295–298 (2003).
17. Mazel, D., Dychinco, B., Webb, V. A. & Davies, J. Antibiotic resistance in the ECOR collection: Integrons and identification of a novel *aad* gene. *Antimicrob. Agents Chemother*. **44**, 1568–1574 (2000).
18. Maynard, C., J. M. *et al*. Antimicrobial resistance genes in enterotoxigenic *Escherichia coli* O149:K91 isolates obtained over a 23-year period from pigs. *Antimicrob. Agents Chemother*. **47**, 3214–3221 (2003).
19. Perreten, V. & Boerlin, P. A new sulfonamide resistance gene (*sul3*) in *Escherichia coli* is widespread in the pig population of Switzerland. *Antimicrob. Agents Chemother*. **47**, 1169–1172 (2003).
20. Pribul, B. R., Festivo, M. L., De Souza, M. M. S. & Rodrigues, D. D. P. Characterization of quinolone resistance in *Salmonella* spp. isolates from food products and human samples in Brazil. *Braz. J. Microbiol*. **47**, 196–201 (2016).
